# Supplementary material for: Reconciling Mining with the Conservation of Cave Biodiversity: A Quantitative Baseline to Help Establish Conservation Priorities
Source: PLoS One. 2016 Dec 20;11(12):e0168348. doi: 10.1371/journal.pone.0168348 (PMC5173368; doi:10.1371/journal.pone.0168348)
Supplement: S1 Dataset — (ZIP) [file pone.0168348.s002.zip › Taxa/Serra Norte/SN_2013/N5S/Lista N5S-12.pdf]

CAVIDADE N5S-0012

Classe	Ordem	Fam/Outros	Gên/Outros	Espécie	S-U	U-S	Zon.
Annelida	Oligochaeta			jovem	8		E/P/A
Arachnida	Acari	Asternoseiidae		sp.84	X		P
Arachnida	Acari	Astigmata		sp.2	X		P
Arachnida	Acari	Ixodidae		jovem	X		P/A
Arachnida	Acari	Mesostigmata		jovem	X		P
Arachnida	Acari	Trombidiidae		sp.15	X		P
Arachnida	Amblypygi	Phrynidae	*Heterophrynus*	*longicornis*	4	X	P
Arachnida	Araneae	Araneidae	*Alpaida*	aff. *negro*	X		E/P
Arachnida	Araneae	Corinnidae	*Tupirinna*	sp.1	5		P
Arachnida	Araneae	Ctenidae		jovem	6	X	P/A
Arachnida	Araneae	Drymusidae	*Drymusa*	*spelunca*	X	X	P
Arachnida	Araneae	Ochyroceratidae	*Ochyrocera*	sp.3	X		P
Arachnida	Araneae	Ochyroceratidae	*Speocera*	sp.1	X		P/A
Arachnida	Araneae	Oonopidae	aff. *Triaeris*	sp.1	X	X	P
Arachnida	Araneae	Pholcidae	*Leptopholcus*	sp.1	X		P
Arachnida	Araneae	Pholcidae	*Mesabolivar*	*eberhard*		X	
Arachnida	Araneae	Pholcidae	*Mesabolivar*	sp.1	X		A
Arachnida	Araneae	Pholcidae	Ninetinae	sp.1	X		P
Arachnida	Araneae	Pisauridae		jovem	1		E
Arachnida	Araneae	Prodidomidae		jovem	X		P
Arachnida	Araneae	Salticidae		sp.6	X		P
Arachnida	Araneae	Salticidae		sp.7	X		E/P
Arachnida	Araneae	Scytodidae	*Scytodes*	jovem	1		P
Arachnida	Araneae	Segestriidae	*Ariadna*	sp.1	X		E/P
Arachnida	Araneae	Theraphosidae	aff. *Holothele*	sp.1	13	X	E/P
Arachnida	Araneae	Theridiidae	*Theridion*	sp.1	X		P
Arachnida	Araneae	Theridiidae	*Theridion*	sp.2	X		P
Arachnida	Araneae	Theridiidae	*Thymoites*	sp.1	X		P
Arachnida	Araneae	Theridiidae		sp.		X	
Arachnida	Araneae	Theridiosomatidae	*Plato*	sp.1	X	X	P/A
Arachnida	Opiliones	Cosmetidae		sp.		X	
Arachnida	Opiliones	Escadabiidae		sp.2	X	X	P/A
Arachnida	Opiliones	Manaosbiidae	*Saramacia*	sp.1	1		E/P
Arachnida	Opiliones	Sclerosomatidae	*Prionostema*	sp.1	13		E/P/A
Arachnida	Opiliones	Stygnidae	*Protimesius*	*gracilis*	3		P
Arachnida	Palpigradi	Eukoeneniidae	*Allokoenenia*	sp.1	X		P
Arachnida	Pseudoscorpiones	Chernetidae	*Spelaeochernes*	sp.1	X	X	E/P/A
Arachnida	Pseudoscorpiones	Chthoniidae	*Pseudochthonius*	sp.1	X		P
Arachnida	Schizomida	Hubbardiidae	aff. *Rowlandius*	sp.1	X		P
Chilopoda	Scutigeromorpha	Pselliodidae	*Sphendononema*	jovem	3		E/P
Diplopoda	Polydesmida	Chelodesmidae		sp.3	1		P
Diplopoda	Polydesmida	Pyrgodesmidae		jovem	X		P
Diplopoda	Spirostreptida	Pseudonannolenidae	*Pseudonannolene*	jovem	3		P
Entognatha	Collembola	Cyphoderidae		sp.1	X		P
Entognatha	Collembola	Isotomidae		sp.1	X		P
Entognatha	Collembola	Paronellidae		sp.1	X		P
Entognatha	Collembola	Paronellidae		sp.4	X		P
Entognatha	Diplura	Campodeidae		sp.1	X		E/P
Entognatha	Diplura	Japygidae		sp.1	X		P

Insecta	Blattodea	Blattellidae		sp.1	21	X	E/P/A
Insecta	Coleoptera	Scydmaenidae		sp.8	X		P
Insecta	Coleoptera	Staphylinidae		sp.15	X		P
Insecta	Diptera	Bombyliidae		sp.	X		P
Insecta	Diptera	Chironomidae		sp.	X		P
Insecta	Diptera	Psychodidae	Phlebotominae	sp.	X		P
Insecta	Diptera	Sphaeroceridae		sp.	X		P
Insecta	Diptera	Tipulidae	Tipulinae	sp.	X		E/P
Insecta	Heteroptera	Cydnidae		sp.		X	
Insecta	Heteroptera	Dipsocoroidea		jovem	X		P
Insecta	Heteroptera	Reduviidae	Emesinae	sp.2	2		P
Insecta	Heteroptera	Reduviidae	Reduviinae	sp.	3		E
Insecta	Heteroptera	Veliidae	*Paravelia*	sp.	X		P
Insecta	Homoptera	Cixiidae		jovem	X	X	P
Insecta	Hymenoptera	Diapriidae		sp.3	X		P
Insecta	Hymenoptera	Formicidae	*Camponotus*	sp.		X	
Insecta	Hymenoptera	Formicidae	*Eurhopalothrix*	sp.1	X		P
Insecta	Hymenoptera	Formicidae	*Hypoponera*	sp.1	X		E/P
Insecta	Hymenoptera	Formicidae	*Pachycondyla*	sp.2	X		P
Insecta	Hymenoptera	Formicidae	*Paratrechina*	sp.2	X	X	E/P
Insecta	Hymenoptera	Formicidae	*Pheidole*	sp.2	X		P
Insecta	Hymenoptera	Formicidae	*Pheidole*	sp.3	X		P
Insecta	Hymenoptera	Formicidae	*Pseudomyrmex*	sp.1	X		P
Insecta	Isoptera	Termitidae	*Syntermes*	sp.	X	X	E
Insecta	Lepidoptera	Gelechioidea		sp.	X		P
Insecta	Lepidoptera	Noctuoidea		sp.5	1		E/P
Insecta	Lepidoptera	Tineoidea		sp.		X	
Insecta	Lepidoptera	Tineoidea		sp.1	X		P
Insecta	Lepidoptera	Tineoidea		sp.3	X		P
Insecta	Orthoptera	Gryllidae		sp.	2		P
Insecta	Orthoptera	Phalangopsidae	*Paraclodes*	sp.	11		E/P
Insecta	Orthoptera	Phalangopsidae	*Phalangopsis*	sp.	55	X	P
Insecta	Psocoptera	Epipsocidae	*Epipsocus*	sp.1	X		P
Insecta	Psocoptera	Epipsocidae	*Epipsocus*	sp.2	X		P/A
Insecta	Psocoptera	Epipsocidae	*Mesepipsocus*	sp.1	X		P
Insecta	Psocoptera	Manicapsocidae	*Nothoentomum*	sp.1	X		P
Insecta	Thysanura	Nicoletiidae		sp.1	X		P
Gastropoda	Pulmonata	Subulinidae	*Pseudopeas*	sp.	X	X	A
Gastropoda	Pulmonata	Systrophiidae	*Happia*	sp.		X	
Symphyla		Scutigerellidae		sp.	X		P
Amphibia	Anura	Leptodactylidae	*Leptodactylus*	cf. *vastus*	1	X	P
Amphibia	Anura	Strabomantidae	*Pristimantis*	cf. *fenestratus*	4		E/P
Mammalia	Chiroptera	Phyllostomidae	*Anoura*	*caudifer*	2		P
Mammalia	Chiroptera	Phyllostomidae	*Carollia*	*perspicillata*	3		P
Mammalia	Chiroptera	Phyllostomidae	*Glossophaga*	*soricina*	1		P
